# Operation in Breast Cancer

**Published:** 1902-05-24

**Authors:** 


### OPERATION IN BREAST CANCER.

At the last meeting of the Royal Medical and Chirurgical Society Mr. Thomas Bryant brought forward an analysis of 46 cases of cancer of the breast which had been operated upon and had survived the operation from five to 32 years. His aim in bringing forward these cases was apparently two-fold: on the one hand he wished to show that the three years' freedom from recurrence after a primary operation "which has been so dogmatically laid down as a significant indication of a cure of cancerous disease" is an unreliable and misleading test, and on the other to show that a less severe operation than the one which has lately been advocated in the treatment of this disease is sufficient for all practical purposes. Certainly the cases which he gave are sufficiently striking. In 17 there had been no recurrence five or more years after the primary operation. Of these 13 are now alive at periods varying from five to 16 years afterwards, and four have died at 5, 20, 14, and 13 years respectively after operation. In 29 cases recurrence took place, either in the site of operation or elsewhere, at periods ranging from one to 32 years after the primary operation. In estimating the value of the particular form of operation employed the seat of the recurrence is a matter of much importance. This was in and about the scar of the original operation in 14 cases; in the scar and axilla in only one case; in the sternum in three cases; in the second breast in 10 cases; and in five of the latter the scar of the first operation was also involved. Mr. Bryant asks those surgeons who advocate the clearing out of the axilla of all lymphoid tissue as a rule of practice in every case to consider these figures; in only one case was the axilla cleared out in an operation for a recurrent affection, and it is not his custom to clear it out at all. By way of summary Mr. Bryant expresses his conviction that the results of operation for cancer, whether of the breast or elsewhere, would be much better than they are now if they could always be undertaken during the early development of the disease; that every breast tumour neither clearly inflammatory nor encapsuled which seems to involve gland tissue should be at once explored, and removed if found to be cancerous with the whole gland; and that recurrent growths when localised should be similarly treated. The matter discussed by Mr. Bryant in this paper is one of extreme interest to the practical surgeon. A careful consideration, however, of the facts brought forward leads us to the conclusion that their teaching lies far more in the direction of early operation than of lessening the extent of the operation. It is sufficiently obvious that all Mr. Bryant's successes would have still been successes if they had been operated on by Halsted's method, and perhaps a few more; that is of course if one could eliminate the immediate risk of the operation. The question then veers round to that of the comparative risks of the two styles of operation, a matter which was not dealt with in the paper. But as to the advantage of early operation the case is absolute, for in regard to 17 cases in which no recurrence was observed we are told that the disease was, in most of them, in an early stage of development when submitted to operation. There was a lump in the breast without skin implication or lymphatic glandular enlargement; the question arose as to the lump being due to the presence of a cyst or early cancerous infiltration; an exploratory incision was made into the lump, and when cancer was recognised the gland was removed. "Under these circumstances," as Mr. Bryant very properly remarks, "the good results which have been recorded are to be explained." And that, we think, is the teaching of this most interesting paper.

